# Analysis of medication therapy discontinuation orders in new electronic prescriptions and opportunities for implementing CancelRx

**DOI:** 10.1093/jamia/ocy100

**Published:** 2018-08-07

**Authors:** Yuze Yang, Stacy Ward-Charlerie, Nitu Kashyap, Richelle DeMayo, Thomas Agresta, James Green

**Affiliations:** 1 Surescripts LLC, Arlington, Virginia, USA; 2 Clinical Informatics, Yale New Haven Health System, New Haven, Connecticut, USA; 3 Connecticut Children’s Medical Center, Hartford, Connecticut, USA; 4 Clinical Informatics, Farmington, Connecticut, USA; 5 Connecticut Institute for Primary Care Innovation, Hartford, Connecticut, USA; 6 University of Connecticut, Storrs, Connecticut, USA

**Keywords:** E-prescribing, electronic prescription cancellation, patient safety, quality, CancelRx, medication safety

## Abstract

**Objective:**

To illustrate the need for wider implementation of the CancelRx message by quantifying and characterizing the inappropriate usage of new electronic prescription (NewRx) messages for communicating discontinuation instructions to pharmacies.

**Materials and Methods:**

A retrospective analysis on a nationally representative random sample of 1 400 000 NewRx messages transmitted over 7 days to identify e-prescriptions containing medication discontinuation instructions in NewRx text fields. A vocabulary of search terms signifying cancellation instructions was formulated and then iteratively refined. True-positives were subsequently identified programmatically and through manual reviews. Two independent reviewers identified incidences in which these instructions were associated with high-alert or look-alike-sound-like (LASA) medications.

**Results:**

We identified 9735 (0.7% of the total) NewRx messages containing prescription cancellation instructions with 78.5% observed in the Notes field; 35.3% of identified NewRxs were associated with high-alert or LASA medications. The most prevalent cancellation instruction types were medication strength or dosage changes (39.3%) and alternative therapy replacement orders (39.0%).

**Discussion:**

While the incidence of prescribers using the NewRx to transmit cancellation instructions was low, their transmission in NewRx fields not intended to accommodate such information can produce significant potential patient safety concerns, such as duplicate or inaccurate therapies. These findings reveal the need for wider industry adoption of the CancelRx message by electronic health record (EHR) and pharmacy systems, along with clearer guidance and improved end-user training, particularly as states increasingly mandate electronic prescribing of controlled substances.

**Conclusion:**

Encouraging the use of CancelRx and reducing the misuse of NewRx fields would reduce workflow disruptions and unnecessary risks to patient safety.

## BACKGROUND AND SIGNIFICANCE

Propelled by several legislative mandates including the Health Information Technology and Clinical Health (HITECH) Act, the Medicare Improvements for Patients and Providers Act (MIPPA), as well as several billion dollars endowed by the Centers for Medicaid and Medicare Services (CMS) in incentives and infrastructure developments to meet Meaningful Use requirements, electronic prescribing (e-prescribing) has become one of the most widely used components of the U.S. healthcare information technology infrastructure due to its prospects of producing increased efficiency, reduced costs, and improved care quality.^1–5^,^6^,^7^ Electronic prescribing of controlled substances (EPCS), legal in all 50 states, in particular, continues to grow, with 4 states having passed legislation mandating its use. As with any technology, the benefits from e-prescribing are directly dependent on the manner in which it is implemented and utilized. Prior research has demonstrated that the inappropriate implementation and utilization of e-prescribing technology can elevate the risks for certain errors or even introduce new sources of error.^8–11^ Most physicians have the ability to both electronically create and discontinue orders related to every aspect of the patient’s care in their typical electronic health record (EHR) system workflows, ranging from labs and diagnostics to specific medication regimens. They subsequently also expect to have their instructions, whether they be the original order or a subsequent change or cancellation order, received and executed in a timely manner. However, prescribers cancelling or changing a medication order electronically may not be aware that unless both their EHR system and the dispensing pharmacy have implemented the CancelRx transaction functionality, changes made to the patient’s medication list will be limited to their own EHR and will not be automatically communicated to the pharmacy. In these instances, prescribers must rely on other means of communication, such as phone calls or faxes, to convey intended changes in a patient’s regimen to the pharmacy to prevent medication therapies deemed unnecessary or inappropriate from being dispensed to the patient.

**Figure 1.**
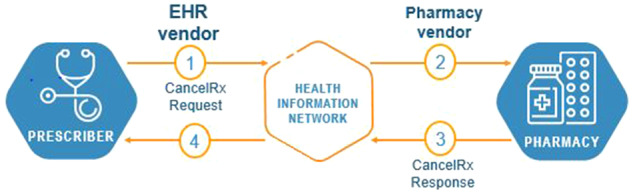
CancelRx transaction process.

**Figure 2.**
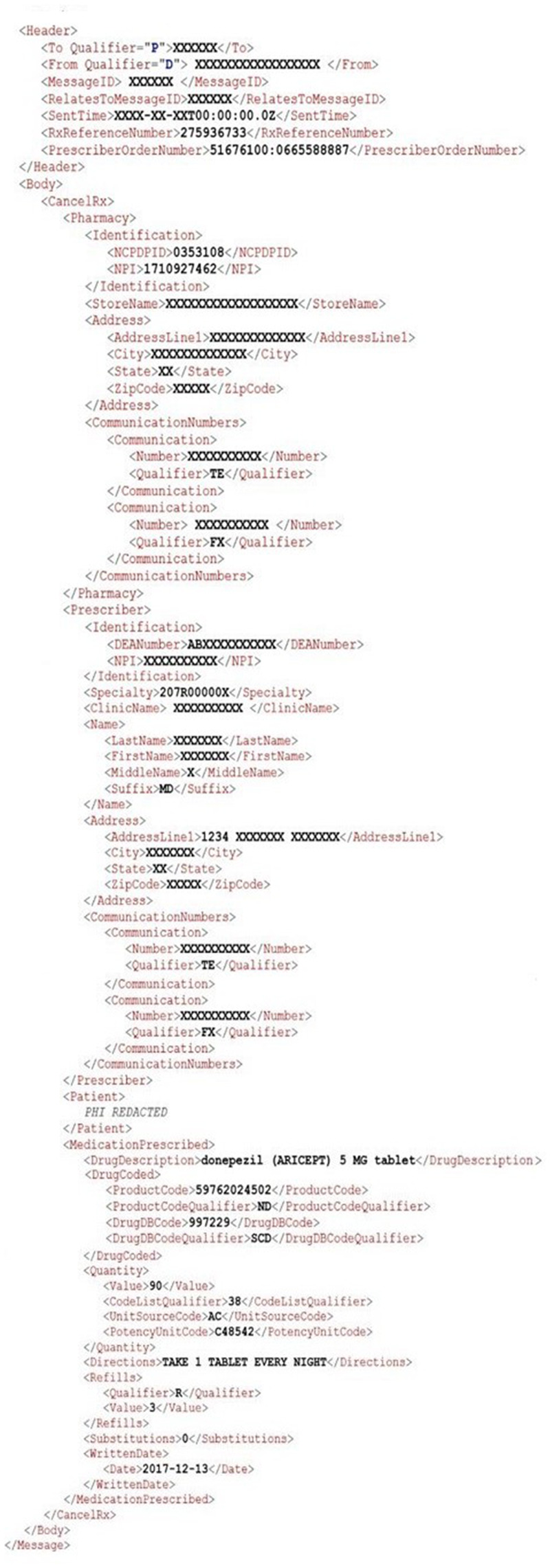
Example XML of a CancelRx request message.

Since the ability for providers to communicate instructions to pharmacies to cancel future dispensing of certain medications to the patient is a vital aspect of efficient and safe patient care, the CancelRx transaction was established as a separate electronic message distinct from the new electronic prescription (NewRx) message by the National Council for Prescription Drug Programs (NCPDP) SCRIPT Standard version 10.6 (Figures 1 and 2).^12^,^13^ This transaction is initiated by the EHR system when a prescriber or prescriber agent discontinues a medication order or prescription in a patient’s record. The discontinuation may stem from any number of reasons, such as an order entry error, a desire to change one therapy to another more efficacious option or a more cost-effective formulary alternative, or a replacement regimen, such as a product strength adjustment, eg warfarin 3 mg to 5 mg tablets, or dosing adjustment, etc.

However, despite having been available since 2010, this transaction is an optional functionality for both EHR systems and pharmacies and was not explicitly incentivized by Meaningful Use, and thus has experienced low adoption and suboptimal utilization. The low adoption by EHR systems and pharmacy systems is perhaps justly attributable to hesitancy from each side until a critical mass of adoption by the other justifies the substantial implementation efforts to reach full realization of CancelRx’s benefits. Due to the lack of end-user knowledge and the technology implementation gap for intended functions, some prescribers may opt to utilize elements in NewRx messages instead of the specific CancelRx message to communicate orders for discontinuation or alteration of medication regimens. In the NewRx, prescribers are able to manually enter information in certain free-text fields, eg, the Notes field, though such actions can cause considerable downstream effects on pharmacy workflows, especially if the free-text field is cluttered with other information and is not expected by pharmacists to contain essential cancellation instructions.^14^ Communication of such information in inappropriate fields of the NewRx is a contravention of the Meaningful Use requirements intended to promote efficient workflows and high-quality care.^15^ Furthermore, it introduces medication ordering and dispensing errors with dangerous risks to patient safety when the discontinuation instructions are not effectively conveyed and are missed by pharmacists and by other prescribers who may be retrieving outside medication records via health information exchanges. Prior studies have illustrated that patients who continue to receive duplicate or unnecessary medication therapies due to medication ordering or dispensing errors are at higher risks for adverse drug events and patient outcomes, as well as excessive costs from unnecessary dispensings and refills.^16–18^ The problem is further compounded when multiple providers manage a patient’s drug therapy, especially during transitions of care and among different EHR systems.

## OBJECTIVES

The objectives of this study were to quantify the incidence rate of the inappropriate usage of the NewRx transaction for communicating therapy regimen changes or discontinuation orders, characterize the various types of instructions communicated, and identify instances specifically involving high-alert or look-alike-sound-alike (LASA) medications, according to the Institute for Safe Medication Practices (ISMP), which may be more prone to be ordered erroneously.^19^,^20^ The findings may subsequently be used to drive the adoption, optimization, and familiarity of the CancelRx transaction, as well as formulation of best-practice recommendations for improving e-prescribing workflows and patient safety.

## METHODS

We conducted a retrospective analysis of 3 specific fields in a nationally representative random sample of 1 400 000 NewRx messages transmitted by ambulatory care prescribers through a national health information network to community pharmacies across the United States from November 6 to November 12, 2016. This sample was drawn by randomly selecting 200 000 NewRx messages each day for 7 days out of an average daily network total of 4.1 million NewRxs, and was calculated to be representative with a margin of error of 0.8% at a confidence level of 99.9%.^21^ The specific data elements analyzed from each NewRx included the provider identification numbers, Drug Description, Sig, and Notes fields. No patient-specific information was made available to the investigation team. Consequently, when the study proposal and design was submitted to a third-party institutional review board (IRB) for approval, the IRB determined this study to be exempted from review.^22^ Study analysis was performed from January 9, 2017, to August 24, 2017, in 2 phases.

### Phase 1: Identification of cancellation or therapy change orders in NewRx fields

To identify instances in which a prescriber entered orders to discontinue or change a medication, we searched for specific text strings in 3 fields of the NewRx transaction that can allow for free text—Drug Description, Directions (Sig), and Notes. The formulation of the search terms was created through an iterative process, in which the vocabulary of terms was refined through repeated programmatic queries using structured query language (SQL) followed by subsequent manual reviews to identify instances of false positives and false negatives. In each iteration, a random sample of 200 000 NewRxs was extracted and queried to identify any messages in which any of the search terms appeared in any of the 3 aforementioned fields. Two residency-trained pharmacists (Y.Y. and S.W.C) then manually reviewed the sample, with one pharmacist specifically analyzing the initial positive hits for false positives, and the other specifically analyzing the remaining sample for false negatives. This process of refining the list of search terms and expanding another set of exclusion terms to eliminate false-positive hits was repeated until an iteration was reached for which the manual reviews produced no new false positives or false negatives.

In the initial iteration of querying NewRxs for cancellation instructions, a random sample of 200 000 messages transmitted in 1 day was extracted and queried using 18 search terms. Examples of search terms included text strings such as “*DC*,” “*stop*,” “*discontinue*,” or “*change dose*,” etc. The query yielded 4579 NewRxs identified as having possible cancellation instructions. Following the review by a pharmacist, 4106 e-prescriptions were identified as false positives, leaving 473 e-prescriptions designated as true-positive hits. For example, while querying the text fields for the search term “*DC*” initially produced several positive hits, upon manual review, many false positives were identified, such as “*Adcirca*” in Drug Description fields or “*Renewal Request From: Medco mail order*” in Notes fields. Based on this review, 34 terms were added to an exclusion vocabulary list to prevent false positives in future querying iterations. In addition, 43 e-prescriptions were identified as false negatives, consisting of newly identified text string variations, synonyms, and syntactic variations of phrases or terminologies for cancelling or replacing medication regimens that were not originally included in the initial search term vocabulary. Consequently, 6 new search terms were added, 9 search terms were edited (eg, “*this replaces*” changed to simply “*replace*”; “*discard previous*” changed to simply “*discard*,” etc.), and 1 study term word was removed due to its redundancy, resulting in an updated vocabulary of 24 search terms.

In the second iteration, another random sample of 200 000 e-prescriptions was extracted from a different day. The sample was queried to identify those e-prescriptions containing any of the updated inclusion vocabulary of search terms while eliminating any hits for the list of exclusionary search terms. Using the 2 vocabulary sets, 4059 NewRxs were identified as containing possible cancel messages in at least 1 field, and of these, 1094 e-prescriptions were classified as false positives based on the study exclusion list, leaving 2965 e-prescriptions as possible true-positive hits. The manual review identified 12 new search terms being added to account for these false positives, along with 9 new exclusion terms identified from manually reviewing false negatives. Newly identified terms from these e-prescriptions were subsequently added to further refine the vocabulary of search terms for inclusion in subsequent iterations.

In a third iteration of querying another random sample of 200 000 NewRxs, pharmacist reviewers identified an additional 5 new search terms from false negatives, and another 9 new exclusion terms in false positives. Upon updating the search vocabulary and running a fourth query including the new keywords, no additional false positives or false negatives were identified in the third manual review. Thus, the search vocabulary was considered finalized by the study investigators and consisted of 41 search terms and 52 exclusion terms (Table 1).

**Table 1.** Finalized vocabulary of search terms and exclusion terms

FINAL SEARCH TERMS N = 41		FINAL EXCLUSION TERMS N = 52	
*Cancel*	*in place of*	*ADC*	*IDC*
*Change Dose*	*Incorrect*	*Adcirca*	*ID# DCHH*
*Change in Dose*	*Increase dose*	*And stop*	*knee replacement*
*Changed*	*Increased dose*	*Antacid/calcium*	*may stop*
*Corrected*	*Increase in dose*	*Avoid*	*MEDCO*
*Correcting*	*Mistake*	*Bidcc*	*NDC*
*Correction*	*no longer taking*	*Can stop*	*nystop*
*Cxl*	*Note change from*	*CDC*	*Once current supply is gone, will DC*
*d/c*	*Note change in*	*COLD/COUGH*	*Ordering provider changed*
*d\c*	*Replace*	*correction scale*	*plus correction*
*Dc*	*Replaced*	*d/c if*	*Postop*
*Decrease dose*	*sent in error*	*DCTR*	*stop after*
*Decreased dose*	*Stop*	*decrease dose by 20% when physically active*	*stop for*
*Decrease in dose*	*therapy end*	*discard diabetic sharps*	*Stop for 1 week*
*Delete*	*void*	*DISCARD EXCESS*	*stop if*
*discard*		*discontinue if*	*Stop x1 week*
*discontinue*		*do not delete the subsequent prescriptions*	*then dc, then d/c*
*disregard*		*do not discontinue*	*then discontinue*
*Dosage change*		*do not stop*	*then stop*
*Dose adjusted*		*For calcium replacement*	*udc*
*Dose Change*		*For iron replacement*	*Until directed to Stop*
*Dose decrease*		*For replacement of*	*until instructed to stop*
*Dose decreased*		*For thyroid replacement*	*USE AND DISCARD*
*Dose increase*		*For Vitamin D replacement*	*void after*
*Dose increased*		*Headache*	*void in XX days*
*Ignore*		*I stop, Istop, i-stop*	*void where prohibited*

Following the finalization of the search vocabularies, a SQL query was performed on the study sample of 1 400 000 NewRxs, consisting of 200 000 NewRxs randomly sampled from each of the 7 days, which excluded any instances of text strings containing any exclusionary terms. Subsequently, a final manual review was conducted on the NewRxs identified through the query. No additional false positives were detected in these NewRxs, thereby leaving only true-positive hits. These true positives were analyzed for their distribution in the 3 NewRx fields and provided to 2 independent reviewers for additional evaluation in Phase 2.

### Phase 2: Categorization of medication cancellation orders and identification of associations with high-alert or LASA medications

In the second phase, 2 PharmD candidates completing Advanced Pharmacy Practice Experience rotations, each with over 3 years of experience as pharmacy technicians in community settings, manually reviewed and classified the various types of cancellation orders identified in Phase 1 as true-positive hits following a classification scheme created by the principal investigator (Table 2). In addition, reviewers identified instances in which the true-positive hits were associated with high-alert or LASA medications as defined by by ISMP. Each reviewer received training from the principal investigator, followed by individual assessments to ensure proficiency with the categorization review.

**Table 2.** Classification scheme for types of cancellation or therapy change orders

Classification code	Cancellation or therapy change order reason type
**A**	Alternate therapy, formulary change, or drug selection change for a more efficacious or therapeutically appropriate product
**B**	Brand or manufacturer-related instructions; eg, stop brand product, change to generic, discontinuation due to manufacturer issues, etc.
**C**	Completion of therapy; condition resolved, prescribed course no longer necessary
**D**	Dose change, eg, patient switching from a 10 mg product to a 20 mg product, or switching from 3 mg and 5 mg Coumadin to 4 mg and 6 mg Coumadin, etc.
**E**	Erroneous order – drug regimen ordered by mistake, generic instructions to void or disregard previous order(s) with no specific rationale
**Other**	Other type of instruction not pertaining to any of the above reason categories

Each reviewer’s assigned classifications were subsequently reconciled, with any discrepancies receiving final adjudications and classifications by the pharmacist primary investigator. Their inter-rater reliability was assessed by calculating the Cohen’s Kappa (κ) coefficient.^23^

## RESULTS

Across the 7-day sampling period, the 1 400 000 NewRxs were transmitted through Surescripts, a national health information network, by 410 591 prescribers using 734 EHR systems or e-prescribing software applications across all 50 states, District of Columbia, and 4 U.S. territories to 64 363 pharmacies.^24^ The text-mining and sample querying process yielded 9735 (0.7%) NewRxs that contained true-positive hits for medication discontinuation or therapy change orders in at least 1 of the 3 text fields. As detailed in Table 3, the field most frequently used by prescribers to enter cancellation or therapy change instructions was the Notes field (78.5%), followed by the Sig field (21.4%), and finally the Drug Description field (0.1%).

**Table 3.** Medication cancellation or change orders identified in NewRx text fields

Prescription field	N (%)	Examples^a^
**Drug description**	**8 (0.1%)**	*Trazodone 50 mg tablet discontinue Remeron*
		*rx benzonatate (TESSALON) 100 mg capsule (DC MED)*
		*Vistaril oral 25 mg capsule discontinued*
		*Cancel Brillinta order*
**Sig**	**2122 (21.4%)**	Take 2 tablets by oral route two times per day. **D/C all other Metformin RX**
		*1 tab(s) po bid, Instr: stop the LA version*
		*50 MG PO 1TAB daily Cancel previous scripts; Decrease dose to 1 TAB daily*
		*i tab po qhs—this dose replaces the prior 25 mg dose—d/c that dose*
**Notes**	**7765 (78.5%)**	*This is a dose increase. Discontinue 10 mg. dose.*
		*This Rx is a change. Please discontinue previous Vistaril 50MG Capsule from 10/14/2016.*
		*Please d/c rx for #60 sent a few minutes ago. Thanks.*
		*Stop any tetracyclin computer states he is on and don't find it*
**TOTAL**	**9895**^b^**(100%)**	

a All examples display the exact text strings that appeared in the original e-prescription message.

b Some e-prescriptions contained cancellation instructions in multiple text fields.

Based on the classification of the types and reasons for cancellation orders, the most frequently observed cancellation orders were category “D” (39.3%), ie, dosage change-related orders, and category “A” (39.0%), ie, alternative drug therapy orders prescribed to replace previous medication(s). Agreement between the 2 reviewers was strong, with a resultant κ = 0.82. The distribution of categorized cancellation orders is summarized in Table 4.

**Table 4.** Classification of types of cancellation or therapy change orders

Classification code	Drug description	Sig	Notes	N	Example 1^a^	Example 2
A	1	1095	2804	3156 (39.0%)	*To replace rx for ambien due to ineffectiveness*	*Take 1 Tablet Daily. Take in place of torsemide.*
B	0	2	51	53 (0.5%)	*please dc generic*	*Patient requesting to try generic rather than brand due to cost. Please cancel previous order*
C	6	107	159	272 (2.7%)	*Cipro discontinued.*	*Pt. is no longer on this medication. Please cancel it.*
D	1	846	3079	3926 (39.3%)	*This is a dose increase. Discontinue 10 mg dose*	*Please d/c prior orders for perphenazine. This is a decrease in dose.*
E	0	75	1740	1815 (18.2%)	*Cancel/DC/void all previous orders for this medication.*	*I sent this prescription on 11/04/2016 for once daily. It should be twice daily. Please void the once a day prescriptions and place the twice a day prescriptions on file. Thanks!*
Other	0	8	25	33 (0.3%)	*2 TABS PO QAM (Cancel any other refills)*	*Use 2 Puffs by inhalation 2 times daily. New provider, stop Rx for this medication from previous provider, hold until next fill*
**TOTAL**				9999^b^		

a All examples display the exact text strings that appeared in the original e-prescription message.

b Some e-prescriptions contained multiple types of cancellation instructions in one field.

**Figure 3.**
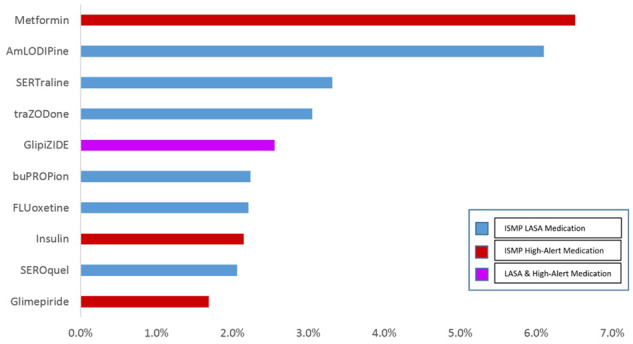
Top 10 high-alert or LASA drugs associated with inappropriate cancellation orders.

Of the 9735 NewRxs with cancellation instructions in free-text fields, 3441 (35.3%) were associated with a high-alert or LASA medication. The most prevalent high-alert drug was the oral hypoglycemic agent metformin, observed in 224 (6.5%) e-prescriptions, followed by LASA medications amlodipine, observed in 210 (6.1%) NewRxs, and sertraline, observed in 114 (3.3%) NewRxs. The distribution of the top 10 most frequently associated high-alert or LASA medications is illustrated in Figure 3. When these 3441 medications were analyzed according to their associations with the various types of cancellation orders, the association with dosage change-related cancellation orders (category D) was most frequently observed in 1546 (44.9%) NewRxs, followed by replacement orders for alternative therapies (category A), observed in 1224 (36.2%) NewRxs, and cancellations related to previous mistakes or erroneous orders (category E), observed in 618 (18.0%) NewRxs.

## DISCUSSION

This study provided novel insights into a healthcare quality-improvement opportunity through the quantification and characterization of incidences in which clinically significant instructions to cancel obsolete or unnecessary medication therapies were communicated sub-optimally as free text in various fields of the NewRx message. Despite the low incidence rate of 0.7%, the actual volume of 9735 instances involving misuse of the NewRx message to communicate cancellation orders or medication therapy regimen change instructions in text fields that were not intended to accommodate such information according to the NCPDP SCRIPT Standard presents a noteworthy concern and a clear use case for CancelRx. This volume exemplified 9735 additional opportunities in a single week for preventing risks that jeopardize the quality and safety of patient care, as the inappropriate use of the NewRx poses substantial workflow challenges at the pharmacy and opportunities for confusion, misinterpretation, or inappropriate continuations of obsolete or unnecessary medications when critical instructions are obscured and overlooked. This rate of 0.7% of NewRx that were inappropriately used to convey cancellations that should have been instead conveyed through CancelRx messages in a single week may be extrapolated to over 9.8 million additional opportunities for correctly utilizing CancelRx each year.^23^ The Notes field in particular has proven to be an attractive field for prescribers to free-text myriad different information—some appropriate that actually warrants the utilization of the field, but much inappropriate or irrelevant as well.^13^ Considerable workflow inefficiencies and patient safety risks are introduced when the field is cluttered with a variety of information, eg, “*d/c lexapro 5 mg Savings for Non-Covered Medications Claims: BIN: XXXX, PCN: BNRX, GROUP: DFSTT, Patient ID: 10-Digit Phone; Questions: YourRx 800-577-6484*.” Not only is such a string difficult to read in an efficient manner, but repeated encounters with such disorderly text in cluttered fields that obscure potentially critical clinical information may eventually cause desensitization in pharmacists, who must often process considerable amounts of information from prescriptions rapidly in their usual busy workflows. Moreover, the pharmacies are not the only ones that experience the resultant impact from downstream workflow inefficiencies and errors. If the cancellation instructions are missed and the obsolete therapy continues to be dispensed, the workflow challenges circle back to the prescriber side again, if the patient is admitted back to the hospital and a provider must reconcile the patient’s therapeutic regimens. The mistakes propagated through the suboptimal communication of the original cancellation instruction may continue to be reflected in the patient’s medication history and dispensing records from the pharmacy, thereby causing discrepancies with the hospital’s records and confusion during reconciliation. Thus, the transmission of cancellation orders in NewRx text fields needlessly adds to the difficulties in distinguishing truly clinically significant instructions and to increased safety risks of patients continuing to receive medications that are no longer therapeutically appropriate or necessary—a danger that may persist indefinitely until caught and corrected.

Unsurprisingly, a majority of these cancellation orders were related to discontinuations for a previous dosage or strength of a medication and substitution of a new dosage strength or for a new medication entirely. While the CancelRx transaction supports the communication of the prescriber’s intent to discontinue a previous therapy or dosing instructions for a medication, the intent to replace the regimen with a new one or with a new dosage is also reliant on the subsequent NewRx order that follows the CancelRx and contains the intended changes. EHR workflow processes would therefore need to ensure that both the CancelRx and subsequent NewRx for the new regimen accompany one another with clear linkage conveyed to the pharmacy, and end-user training should ensure prescriber understanding that certain workflows in their EHR involving the adjustment or replacement of a patient’s regimen will often entail first discontinuing the order and then the transmission of a new order. Additionally, the discovery that the third-most prevalent type of cancellation order was related to discontinuing medications ordered in error or with no specifically stated appropriate reason is also noteworthy, especially in conjunction with the finding that 35.3% of cancellation orders were associated with high-alert or LASA medications. These medications have been identified by ISMP as bearing a heightened risk for causing significant patient harm when taken in error or having elevated risks of being ordered erroneously due to being mistaken for similarly sounding medications, respectively.^17^,^18^ The results suggest several opportunities remain for enhancing end-user training and system interface design to improve medication selection accuracy. ISMP-recommended features such as tall-man lettering should be used to help distinguish between similarly sounding drug names, and additional changes in premarketing and postmarketing testing and surveillance of e-prescribing software applications should be leveraged for continual system enhancements.^25^ Furthermore, vendors should consistently deploy user-centered design procedures that solicit continuous feedback from diverse cohorts of prescribers to help drive such system enhancements.

Ultimately, the most obvious and effective solution to the aforementioned suboptimal workflows and patient safety risks is the wider adoption of the CancelRx message by both EHR systems and pharmacies. Despite the fact that CancelRx has existed in the e-prescribing standard since 2010, its implementation by the industry has historically lagged due to hesitant standards adoption, possible perception of certification requirements as being onerous or laborious, long software development and deployment cycles, and competing software development or maintenance priorities.^26^ However, the findings from this study highlight the vital need for the e-prescribing industry to overcome these factors and expedite the adoption and implementation of CancelRx across the industry. This transaction provides a more efficient electronic means of conveying the prescriber’s intentions to discontinue medications than the historic methods of having to call the pharmacy or fax specific instructions. Faxes and phone calls are often considered inconvenient or inefficient, as they require additional steps outside of the typical e-prescribing workflow. Such workflow disruptions may be one reason that prescribers elect to manually free-text cancellation orders using NewRx text fields, as they may feel more convenience from communicating such instructions while they are already in the middle of writing a new separate e-prescription order. However, these prescribers may be unaware of the potential downstream implications and unnecessary risks to patient safety such practices can generate. Therefore, additional end-user education may also be necessary to reduce such behavior and encourage prescribers to pursue only the most effective and appropriate means for communicating their intent, even if that entails inconvenient non-electronic means of phone calls or faxes in situations in which the provider’s EHR system or the recipient pharmacy has not implemented the CancelRx functionality. Additionally, such direct communication with the patient would ensure that the provider’s instructions can be confirmed to be received and clearly understood by all necessary parties.

Fortunately, some EHR systems have begun to increase adoption and implementation of the CancelRx transaction. Possible reasons could stem from the Meaningful Use requirements, or feedback from end-users who are concerned about patient safety ramifications or who find phone calls and faxes to be an inefficient use of their time. However, the transaction is beneficial only if it can be processed and used by a receiver, and the current level of adoption at the pharmacy end remains low. Some pharmacies similarly face hesitation in implementing the functionality to receive and process this new message, as the relatively low industry adoption rate hampers prioritizations for system developments. The low adoption on the pharmacy side may also discourage many EHR vendors, as their prescriber end-users must keep track and follow different workflows accordingly when cancelling medications for patients who fill at pharmacies that can process CancelRx messages and for patients who fill at pharmacies that still rely on calls or faxes. However, the study results highlight an urgent need for a systems-driven and standards-driven solution to eliminate needless additional patient safety risks and inefficient workflow practices. Hence, the benefits from the appropriate utilization of the CancelRx transaction would ultimately outweigh the resource and time requirements from system developments, certification, and end-user education.^27^ In parallel to the EHR adoption of CancelRx, pharmacies should therefore accelerate their own adoption as well. To drive such advancements, pharmacists, pharmacy associations, and regulatory bodies such as state boards of pharmacies should collaborate and establish adoption roadmaps and milestones to attain market saturation that would also spur EHR implementation in a positive feedback loop.

## LIMITATIONS

Several limitations in this study should be noted. First, the sample consisted only of NewRxs transmitted during a 7-day period. Hence, the content observed in these messages may not be entirely representative of all possible information in all e-prescriptions in ambulatory care settings. Second, the study was limited to evaluating inappropriate usage of NewRx messages, but the specific patient outcomes stemming from the receipt and processing of these NewRxs were beyond the scope of investigation. Further studies should therefore be conducted to ascertain the incidence of adverse drug events or significant patient outcomes from missed cancellation instructions and subsequent dispensing of inappropriate or unnecessary therapies to patients by leveraging patient-specific identifiers and their associated medication history data. Third, this study did not analyze or compare the effectiveness of the inappropriate use of the NewRx with the other typical means of communicating cancellation instructions, eg, faxes and phone calls, and whether or not the inappropriate usage of an e-prescription produced more quality or safety concerns or had any benefits over traditional non-electric communications. Finally, this study relied on iterative manual reviews with continuous refinements to establish a list of keywords for mining text strings in NewRx fields. However, there is a possibility that additional search terms could have been missed and should have been added to identify additional true-positive hits. Hence, more complex natural language processing (NLP) algorithms should be explored in future text-mining endeavors, which may produce more comprehensive results.

## CONCLUSIONS

The findings from this study reveal a critical need for increased adoption of the CancelRx transaction, which has been available in the current NCPDP SCRIPT v10.6 Standard for several years. The use of free text in various NewRx fields to communicate instructions to discontinue or change medication therapies presents risk to patient safety as well as numerous possible downstream workflow challenges for pharmacy systems and pharmacists, especially when a significant portion of the NewRxs are associated with high-alert or LASA medications. Higher industry-wide implementation of the CancelRx transaction would therefore benefit both prescribers and pharmacists, improve adherence to Meaningful Use requirements, ensure clearer communication of critical medication regimen information, and enhance the efficiency of both prescriber and pharmacy workflows, thereby ultimately improving safety and quality, and patient care as well.
